# Characteristics of rib fracture patients who require chest computed tomography in the emergency department

**DOI:** 10.1186/s12873-023-00807-9

**Published:** 2023-03-22

**Authors:** Woosik Kim, Juhyun Song, Sungwoo Moon, Jooyeong Kim, Hanjin Cho, Jonghak Park, Sungjin Kim, Sejoong Ahn

**Affiliations:** Department of Emergency Medicine, Korea University Ansan Hospital, 123, Jeokgeum-Ro, Danwon-Gu, Ansan-Si, Gyeonggi-Do 15355 Republic of Korea

**Keywords:** Rib fracture, Chest computed tomography, Intra-thoracic and intra-abdominal injuries, Emergency department

## Abstract

**Background:**

The disadvantages and complications of computed tomography (CT) can be minimized if CT is performed in rib fracture patients with high probability of intra-thoracic and intra-abdominal injuries and CT is omitted in rib fracture patients with low probability of intra-thoracic and intra-abdominal injuries. This study aimed to evaluate the factors that can identify patients with rib fractures with intra-thoracic and intra-abdominal injuries in the emergency department among patients with rib fracture.

**Methods:**

This retrospective observational study included adult patients (age ≥ 18 years) diagnosed with rib fracture on chest radiography prior to chest CT due to blunt chest trauma in the emergency department who underwent chest CT from January 2016 to February 2021. The primary outcomes were intra-thoracic and intra-abdominal injuries that could be identified on a chest CT. Multivariate logistic regression analysis was performed.

**Results:**

Among the characteristics of rib fractures, the number of rib fractures was greater (5.0 [3.0–7.0] vs. 2.0 [1.0–3.0], *p* < 0.001), bilateral rib fractures were frequent (56 [20.1%] vs. 12 [9.8%], *p* = 0.018), and lateral and posterior rib fracture was more frequent (lateral rib fracture: 160 [57.3%] vs. 25 [20.5%], *p* < 0.001; posterior rib fracture: 129 [46.2%] vs. 21 [17.2%], *p* < 0.001), and displacement was more frequent (99 [35.5%] vs. 6 [6.6%], *p* < 0.001) in the group with intra-thoracic and intra-abdominal injuries than in the group with no injury. The number of rib fractures (adjusted odds ratio [aOR], 1.44; 95% confidence interval [CI], 1.16–1.78; *p* = 0.001), lateral rib fracture (aOR, 2.80; 95% CI, 1.32–5.95; *p* = 0.008), and posterior rib fracture (aOR, 3.18; 95% CI, 1.45–6.94; *p* = 0.004) were independently associated with intra-thoracic and intra-abdominal injuries. The optimal cut-off for the number of rib fractures on the outcome was three. The number of rib fractures ≥ 3 (aOR, 3.01; 95% CI, 1.35–6.71; *p* = 0.007) was independently associated with intra-thoracic and intra-abdominal injuries.

**Conclusion:**

In patients with rib fractures due to blunt trauma, those with lateral or posterior rib fractures, those with ≥ 3 rib fractures, and those requiring O_2_ supplementation require chest CT to identify significant intra-thoracic and intra-abdominal injuries in the emergency department.

## Introduction

Rib fractures occur frequently in patients experiencing blunt chest traumas. In the United States, approximately 250,000 patients visit the emergency department annually due to rib fractures. Moreover, the number of patients visiting the emergency department is increasing in the United States [1, 2]. Complications and organ injuries are associated with morbidity and mortality in patients with rib fractures [1, 2].

Chest radiography, ultrasonography, and computed tomography (CT) can be performed in patients with rib fractures in the emergency department [3–5]. However, chest radiography has low sensitivity to detect rib fractures and organ injuries [4, 5]. Ultrasonography examinations require significant time for image acquisition and cause discomfort to injured patients [4, 5]. Injuries in the deep structures of the chest and abdomen are difficult to evaluate by chest radiography and ultrasonography [4–6].

CT is the modality of choice for evaluating rib fractures and associated organ injuries in the emergency department [3–5]. However, radiation exposure risk and cost remain the major disadvantages of CT [7]. Hypersensitivity reactions and acute renal failure are complications of contrast-enhanced CT, used to evaluate organ injury [8]. Undergoing CT of every patient with rib fracture including rib fracture patient with low probability of organ injuries may substantially increase length of stay in the emergency department and may induce overcrowding of the emergency department. Emergency physicians need to balance between accurate diagnosis of organ injuries and disadvantages of CT. Therefore, CT is not always performed in all patients with rib fractures in real world clinical practice in the emergency department.

The disadvantages and complications of computed tomography (CT) can be minimized if CT is performed in rib fracture patients with high probability of intra-thoracic and intra-abdominal injuries and CT is omitted in rib fracture patients with low probability of intra-thoracic and intra-abdominal injuries. Therefore, this study aimed to evaluate the factors that can identify patients with rib fractures with intra-thoracic and intra-abdominal injuries in the emergency department among patients with rib fracture.

## Methods

### Study design and setting

This retrospective observational study was conducted at Korea University Ansan Hospital, the only tertiary academic teaching hospital in Ansan-si, Korea [9]. Approximately 650,000 residents live in Ansan-si, Korea, and 50,000 patients visit the emergency department of Korea University Ansan Hospital annually.

This study was approved by the Institutional Review Board of Korea University (2022AS0117). The institutional review board waived the requirement of informed consent due to the retrospective observational design of the study.

### Definition

In this study, diagnosis of rib fracture was based on chest radiography. Intra-thoracic and intra-abdominal injuries that could be identified on chest CT were obtained. The location of intra-thoracic injury was classified as mediastinum, aorta, diaphragm, lung, pericardium, heart, or thoracic vertebra. The locations of intra-abdominal injuries were classified as liver, spleen, kidney, adrenal gland, pancreas, gut, abdominal vessels, and lumbar vertebra.

Intra-thoracic and intra-abdominal injuries were defined as injuries with an Abbreviated Injury Scale score ≥ 2 in the intra-thoracic and intra-abdominal organs. Significant intra-thoracic and intra-abdominal injuries were defined as intra-thoracic and intra-abdominal injuries except for occult pneumothorax, scanty hemothorax, and small lung contusions that can be only identified on a chest CT (not visible on chest radiography). Occult pneumothorax, scanty hemothorax, small lung contusions, and no injuries were considered nonsignificant intra-thoracic and intra-abdominal injuries.

The mechanism of injury was dichotomized into high-energy and low-energy trauma. High-energy trauma comprised motor vehicle accidents, pedestrian trauma (hit by a motor vehicle), falls of more than 3 m, and industrial trauma. Low-energy trauma include falls within a height level, simple contusions, assault, and unknown trauma mechanisms.

Characteristics of rib fractures include the location (anterior, lateral, and posterior), displacement of more than 50% of the ribs, bilateral rib fractures, and lower rib fractures. A lower rib fracture was defined as a fracture between the 8th and 12th ribs.

### Study population

Adult patients (age ≥ 18 years) diagnosed with rib fracture on chest radiography prior to chest CT due to blunt chest trauma in the emergency department and who underwent chest CT between January 2016 and February 2021 were included in the study. Patients who had a penetrating chest trauma, were age < 18 years, or did not undergo chest CT were excluded. Patients with rib fractures due to cardiopulmonary resuscitation were excluded.

### Data collection

The following data of the patients were extracted from electronic medical records: sex, age, comorbidities, trauma mechanism, administration of antiplatelet or anticoagulant drugs, and initial vital signs upon arrival at the emergency department. Data on management in the emergency department, such as oxygen supplementation, use of mechanical ventilators, and transfusion, were collected. Additionally, the results of chest radiography, chest CT, and injuries to the head, neck, face, extremities, and external wounds were collected. The injury severity score was calculated.

### Outcomes

The primary outcomes were any intra-thoracic and intra-abdominal injuries that could be identified on a chest CT. The secondary outcomes were significant intra-thoracic and intra-abdominal injuries.

### Statistical analysis

Continuous variables are expressed as medians and interquartile ranges or means and standard deviations, according to the distribution of variables. Continuous variables were compared using the Mann − Whitney test or Student’s t-test, as appropriate. Categorical variables are expressed as numbers and percentages and were compared using the chi-square test or Fisher’s exact test.

Multivariable logistic regression analyses were performed to identify independent factors associated with the outcomes. Factors that were significant at the level of 0.1 in univariable logistic regression analyses and those that were well-known risk factors in previous studies were included in the multivariable logistic regression model. Further analyses were performed to identify independent factors associated with intra-thoracic injuries. The Hosmer − Lemeshow test was performed to evaluate the goodness of fit of the models.

Receiver operating characteristic (ROC) curves of the number of rib fractures and outcomes were performed. The area under the curve, sensitivity, and specificity were determined. The Youden index was used to determine the optimal cut-off point. Additional analyses were performed using categorical values according to the optimal cut-off point.

Subgroup analysis was performed among patients without injuries to the head, neck, face, extremities, and external wounds (patients with trauma to the chest and upper abdomen only).

A *p*-value less than 0.05 was considered significant in all statistical analyses. Statistical analyses were performed with R version 4.0.2 software (R Foundation for Statistical Computing, Vienna, Austria).

## Results

**Fig. 1:**
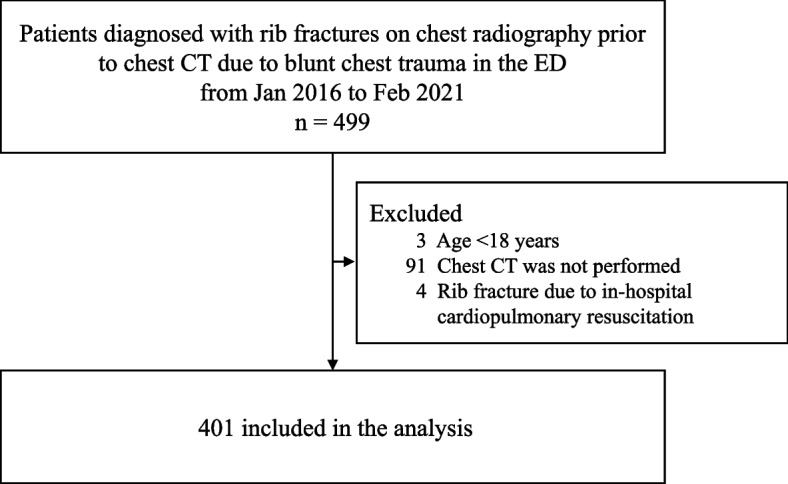
Flowchart of study population. Abbreviation: CT, computed tomography; ED, emergency department

From January 2016 to February 2021, 499 patients were diagnosed in the emergency department with rib fractures on chest radiography prior to chest CT due to blunt chest trauma. Three patients were excluded because they were aged < 18 years, and 91 patients were excluded because chest CT was not performed. Four patients were further excluded as their rib fractures were due to cardiopulmonary resuscitation. Thus, a total of 401 patients were included in this study (Fig. 1).

### Any intra-thoracic and intra-abdominal injuries

Table 1 shows the baseline characteristics of patients with any intra-thoracic and intra-abdominal injuries and no injury. Male patients and trauma mechanisms as high-energy trauma were more frequent in the intra-thoracic and intra-abdominal injury group than in the group with no injury. The group with intra-thoracic and intra-abdominal injuries received O_2_ supplementation, mechanical ventilation, and transfusion more frequently than the group with no injury (all *p* < 0.05). Among the characteristics of rib fractures, the number of rib fractures was higher (5.0 [3.0–7.0] vs. 2.0 [1.0–3.0], *p* < 0.001), bilateral rib fracture was more frequent (56 [20.1%] vs. 12 [9.8%], *p* = 0.018), lateral and posterior rib fracture was more frequent (lateral rib fracture: 160 [57.3%] vs. 25 [20.5%], *p* < 0.001; posterior rib fracture: 129 [46.2%] vs. 21 [17.2%], *p* < 0.001), and displacement was more frequent (99 [35.5%] vs. 6 [6.6%], *p* < 0.001) in the group with intra-thoracic and intra-abdominal injuries than in the group with no injury. Traumatic brain injury, facial bone fractures, and fractures in the extremities were more common in the group with intra-thoracic and intra-abdominal injuries than in the group with no injury (all *p* < 0.05). Meanwhile, comorbidities, medications, and initial vital signs were not significantly different between the groups.

**Table 1:** Baseline characteristics according to any intra-thoracic and intra-abdominal injuries

	No injuries (*N* = 122)	Any intra-thoracic and intra-abdominal injuries (*N* = 279)	*p*-value
Sex			0.009
Female	44 (36.1%)	64 (22.9%)	
Male	78 (63.9%)	215 (77.1%)	
Age	59.0 [49.0–67.0]	57.0 [48.5–66.0]	0.384
**Trauma mechanism**			< 0.001
High-energy trauma	56 (45.9%)	190 (68.1%)	
Low-energy trauma	66 (54.1%)	89 (31.9%)	
**Comorbidities**			
Hypertension	43 (35.2%)	89 (31.9%)	0.589
Diabetes	28 (23.0%)	53 (19.0%)	0.440
Stroke	10 (8.2%)	12 (4.3%)	0.181
Heart disease	3 (2.5%)	12 (4.3%)	0.543
Chronic lung disease	0 (0%)	1 (0.4%)	1.000
Liver disease	2 (1.6%)	3 (1.1%)	1.000
Chronic kidney disease	3 (2.5%)	10 (3.6%)	0.780
**Medication**			
Antiplatelet	11 (9.0%)	21 (7.5%)	0.759
Anticoagulant	1 (0.8%)	7 (2.5%)	0.468
**Initial vital signs**			
Systolic blood pressure (mmHg)	138.0 [123.0–156.0]	136.0 [121.0–152.0]	0.607
Diastolic blood pressure (mmHg)	80.0 [71.0–90.0]	82.0 [72.0–89.0]	0.918
Respiratory rate (/min)	18.0 [18.0–18.0]	18.0 [16.0–20.0]	0.302
Heart rate (/min)	84.0 [78.0–91.0]	86.0 [72.0–96.0]	0.700
Body temperature (℃)	36.7 [36.3–37.1]	36.7 [36.2–37.0]	0.337
**In-ED management**			
O_2_ supplementation	23 (18.9%)	147 (52.7%)	< 0.001
Mechanical ventilator use	2 (1.6%)	21 (7.5%)	0.036
Transfusion	2 (1.6%)	39 (14.0%)	< 0.001
**Rib fracture characteristics**			
Number of rib fractures	2.0 [1.0–3.0]	5.0 [3.0–7.0]	< 0.001
Bilateral rib fracture	12 (9.8%)	56 (20.1%)	0.018
Lower rib fracture	53 (43.4%)	167 (59.9%)	0.003
Location of the rib fracture			
Anterior	30 (24.6%)	80 (28.7%)	0.471
Lateral	25 (20.5%)	160 (57.3%)	< 0.001
Posterior	21 (17.2%)	129 (46.2%)	< 0.001
Displacement	8 (6.6%)	99 (35.5%)	< 0.001
**Other injuries**			
Traumatic brain injury	4 (3.3%)	32 (11.5%)	0.014
Facial bone fracture	1 (0.8%)	20 (7.2%)	0.017
Pelvic bone fracture	5 (4.1%)	21 (7.5%)	0.288
Extremity fracture	8 (6.6%)	45 (16.1%)	0.015
Injury severity score	4.0 [1.0–9.0]	9.0 [9.0–14.0]	< 0.001

Data are expressed as median [IQR] or number (percentage), as appropriate

*Abbreviation*: *ED*, emergency department

### Significant intra-thoracic and intra-abdominal injuries

Table 2 shows the baseline characteristics of significant intra-thoracic and intra-abdominal injuries and without significant injury. The group with significant intra-thoracic and intra-abdominal injuries more received O_2_ supplementation, mechanical ventilation, and transfusion than the group without significant injury (all *p* < 0.05). Among the characteristics of rib fractures, the number of rib fractures was greater (5.0 [3.0–8.0] vs. 3.0 [1.0–4.0], *p* < 0.001) and bilateral rib fractures were frequent (44 [24.2%] vs. 24 [11.0%], *p* = 0.001) in the group with significant intra-thoracic and intra-abdominal injuries than in the group with no significant injury. Anterior, lateral, and posterior rib fractures were more frequent (anterior rib fracture: 61 [33.5%] vs. 49 [22.4%], *p* = 0.017; lateral rib fracture: 99 [54.4%] vs. 86 [39.3%], *p* < 0.001; posterior rib fracture: 100 [54.9%] vs. 50 [22.8%], *p* < 0.001) in the group with significant intra-thoracic and intra-abdominal injuries than in the group with no significant injury. Moreover, displacement was more frequent (77 [42.3%] vs. 30 [13.7%], *p* < 0.001) in the group with significant intra-thoracic and intra-abdominal injuries than in the group with no significant injury. Traumatic brain injury and fractures in the extremities were more frequent in the group with significant intra-thoracic and intra-abdominal injuries than in the group without significant injury (all *p* < 0.05). Meanwhile, comorbidities, medications, and initial vital signs, except heart rate, were not significantly different between the groups.

**Table 2:** Baseline characteristics according to significant intra-thoracic and intra-abdominal injuries

	No significant injuries (*N* = 219)	Significant intra-thoracic and intra-abdominal injuries (*N* = 182)	*p*-value
Sex			0.017
Female	70 (32.0%)	38 (20.9%)	
Male	149 (68.0%)	144 (79.1%)	
Age	58.0 [48.0–67.0]	58.0 [50.0–66.0]	0.661
**Trauma mechanism**			< 0.001
High-energy trauma	115 (52.5%)	131 (72.0%)	
Low-energy trauma	104 (47.5%)	51 (28.0%)	
**Comorbidities**			
Hypertension	77 (35.2%)	55 (30.2%)	0.347
Diabetes	45 (20.5%)	36 (19.8%)	0.948
Stroke	16 (7.3%)	6 (3.3%)	0.125
Heart disease	11 (5.0%)	4 (2.2%)	0.222
Chronic lung disease	1 (0.5%)	0 (0.0%)	1.000
Liver disease	3 (1.4%)	2 (1.1%)	1.000
Chronic kidney disease	7 (3.2%)	6 (3.3%)	1.000
**Medication**			
Antiplatelet	20 (9.1%)	12 (6.6%)	0.454
Anticoagulant	4 (1.8%)	4 (2.2%)	1.000
**Initial vital signs**			
Systolic blood pressure (mmHg)	138.0 [124.0–155.5]	135.0 [118.0–152.0]	0.073
Diastolic blood pressure (mmHg)	82.0 [73.0–90.0]	80.5 [68.0–89.0]	0.161
Respiratory rate (/min)	18.0 [18.0–18.0]	18.0 [16.0–20.0]	0.338
Heart rate (/min)	84.0 [73.0–92.0]	86.0 [74.0–98.0]	0.044
Body temperature (°C)	36.7 [36.3–37.0]	36.6 [36.2–37.0]	0.196
**In-ED management**			
O_2_ supplementation	52 (23.7%)	118 (64.8%)	< 0.001
Mechanical ventilator use	6 (2.7%)	17 (9.3%)	0.009
Transfusion	3 (1.4%)	38 (20.9%)	< 0.001
**Rib fracture characteristics**			
Number of rib fractures	3.0 [1.0–4.0]	5.0 [3.0–8.0]	< 0.001
Bilateral rib fracture	24 (11.0%)	44 (24.2%)	0.001
Lower rib fracture	101 (46.1%)	119 (65.4%)	< 0.001
Location of the rib fracture			
Anterior	49 (22.4%)	61 (33.5%)	0.017
Lateral	86 (39.3%)	99 (54.4%)	0.003
Posterior	50 (22.8%)	100 (54.9%)	< 0.001
Displacement	30 (13.7%)	77 (42.3%)	< 0.001
**Location of intra-thoracic and intra-abdominal injuries**			
Mediastinum	0 (0%)	35 (19.2%)	
Aorta	0 (0%)	6 (3.3%)	
Lung contusion	41 (18.7%)	79 (43.4%)	
Heart	0 (0%)	8 (4.4%)	
Pneumothorax	25 (11.4%)	93 (51.1%)	
Hemothorax	68 (31.1%)	124 (68.1%)	
Diaphragm	0 (0%)	5 (2.7%)	
Liver	0 (0%)	23 (12.6%)	
Spleen	0 (0%)	10 (5.5%)	
Kidney	0 (0%)	9 (4.9%)	
Adrenal gland	0 (0%)	7 (3.8%)	
Gut	0 (0%)	6 (3.3%)	
Pancreas	0 (0%)	6 (3.3%)	
Abdominal vessel	0 (0%)	8 (4.4%)	
Vertebra	0 (0%)	49 (26.9%)	
**Other injuries**			
Traumatic brain injury	12 (5.5%)	24 (13.2%)	0.012
Facial bone fracture	10 (4.6%)	11 (6.0%)	0.663
Pelvic bone fracture	11 (5.0%)	15 (8.2%)	0.272
Extremity fracture	17 (7.8%)	36 (19.8%)	0.001
Injury severity score	9.0 [4.0–9.0]	10.0 [9.0–17.0]	< 0.001

Data are expressed as median [IQR] or number (percentage), as appropriate

*Abbreviation*: *ED*, emergency department

### Multivariable logistic regression analyses

In multivariable logistic regression analysis, age, requirement of O_2_ supplementation, number of rib fractures (adjusted odds ratio [aOR], 1.44; 95% CI, 1.16–1.78; *p* = 0.001), lateral rib fracture (aOR, 2.80; 95% CI, 1.32–5.95; *p* = 0.008), and posterior rib fracture (aOR, 3.18; 95% CI, 1.45–6.94; *p* = 0.004) were independently associated with any intra-thoracic and intra-abdominal injuries. The trauma mechanism, initial vital signs, and injuries at other sites were not independently associated with any intra-thoracic and intra-abdominal injuries (Table 3).

**Table 3:** Results of multivariable logistic regression analysis

	Any intra-thoracic and intra-abdominal injuries			Significant intra-thoracic and intra-abdominal injuries		
Variables	aOR	95% CI	*p*-value	aOR	95% CI	*P*-value
Sex (reference = female)	1.40	0.76–2.56	0.277	1.35	0.75–2.43	0.324
Age	**0.97**	**0.95–0.99**	**0.014**	0.99	0.97–1.01	0.558
High-energy trauma	1.51	0.83–2.76	0.175	1.62	0.93–2.84	0.091
**Initial vital signs**						
Systolic blood pressure	1.01	0.99–1.02	0.398	1.00	0.99–1.01	0.877
Diastolic blood pressure	0.99	0.97–1.02	0.699	1.00	0.97–1.02	0.799
Respiratory rate	0.96	0.90–1.03	0.291	0.96	0.89–1.04	0.372
Heart rate	0.99	0.98–1.01	0.492	1.01	1.00–1.03	0.125
**In-ED management**						
O_2_ supplementation	**3.02**	**1.51–6.04**	**0.002**	**3.66**	**2.08–6.45**	** < 0.001**
Mechanical ventilator use	1.53	0.10–23.65	0.760	**0.14**	**0.02–0.84**	**0.031**
Transfusion	4.87	0.77–30.57	0.092	**13.41**	**2.67–67.39**	**0.002**
**Rib fracture characteristics**						
Number of rib fractures	**1.44**	**1.16–1.78**	**0.001**	**1.33**	**1.12–1.58**	**0.001**
Bilateral rib fracture	0.35	0.12–1.02	0.055	0.42	0.17–1.04	0.062
Lower rib fracture	1.08	0.59–1.99	0.798	1.53	0.86–2.72	0.144
Displacement	1.68	0.65–4.35	0.287	1.29	0.65–2.57	0.465
Anterior rib fracture	0.84	0.34–2.08	0.708	1.13	0.52–2.48	0.754
Lateral rib fracture	**2.80**	**1.32–5.95**	**0.008**	0.67	0.34–1.33	0.250
Posterior rib fracture	**3.18**	**1.45–6.94**	**0.004**	**2.58**	**1.37–4.83**	**0.003**
**Other injuries**						
Traumatic brain injury	0.66	0.14–3.12	0.601	0.90	0.33–2.49	0.841
Facial bone fracture	7.18	0.76–67.88	0.086	1.08	0.35–3.33	0.900
Pelvic bone fracture	0.34	0.07–1.75	0.197	**0.25**	**0.07–0.92**	**0.037**
Extremity fracture	1.79	0.56–5.76	0.325	**2.94**	**1.16–7.46**	**0.023**

Hosmer − Lemeshow test showed *p* > 0.05

*Abbreviation*: *aOR* adjusted odds ratio, *CI* confidence interval, *ED* emergency department

Requirement of O_2_ supplementation, mechanical ventilator use, requirement of transfusion, number of rib fractures (aOR, 1.33; 95% CI, 1.12–1.58; *p* = 0.001), posterior rib fracture (aOR, 2.58; 95% CI, 1.37–4.83; *p* = 0.003), pelvic bone fracture, and extremity fracture were independently associated with significant intra-thoracic and intra-abdominal injuries. The trauma mechanism and initial vital signs were not independently associated with significant intra-thoracic and intra-abdominal injuries (Table 3). Hosmer − Lemeshow test showed a good fit in all models (*p* > 0.05).

### Receiver operation characteristic curve

**Fig. 2:**
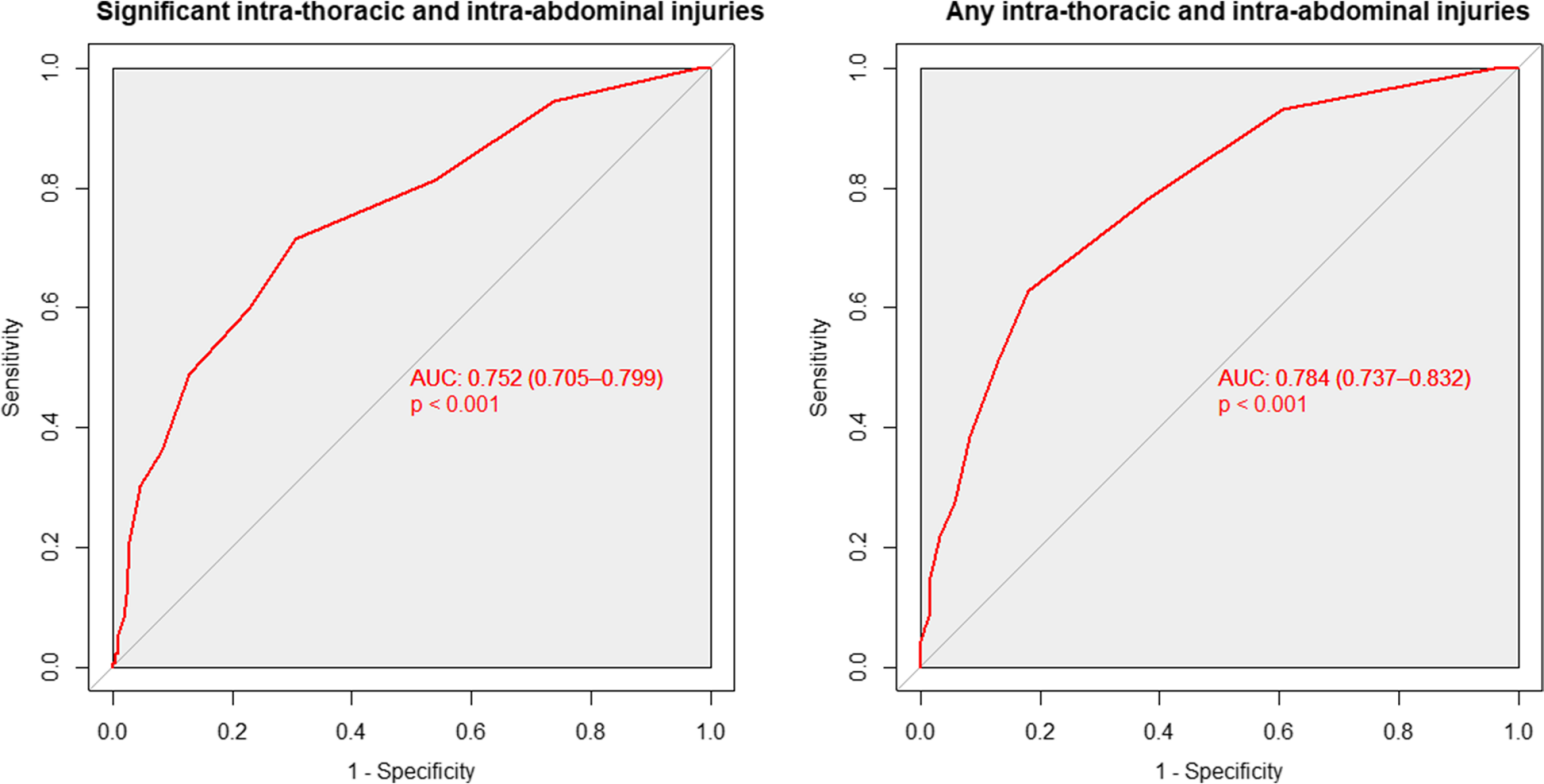
Receiver operation characteristic (ROC) curve of the number of rib fractures as the outcome. ROC curve of the number of rib fractures in those with significant intra-thoracic and intra-abdominal injuries (left). ROC curve of the number of rib fractures in those with intra-thoracic and intra-abdominal injuries (right)

The area under the ROC curve of the number of rib fractures for any intra-thoracic and intra-abdominal injuries was 0.784 (95% CI, 0.737–0.832; *p* < 0.001). The optimal cut-off point was 3, with a sensitivity of 62.7% and specificity of 82.0% (Fig. 2).

The area under the ROC curve of the number of rib fractures for significant intra-thoracic and intra-abdominal injuries was 0.752 (95% CI, 0.705–0.799; *p* < 0.001). The optimal cut-off point was three, with a sensitivity of 71.4% and a specificity of 69.4% (Fig. 2).

### Analysis using the number of rib fractures as a category according to optimal cut-off

Any intra-thoracic and intra-abdominal injuries and significant intra-thoracic and intra-abdominal injuries were more frequent in the group with ≥ 3 rib fractures than in the group with < 3 rib fractures (175 [88.8%] vs. 104 [51.0%], *p* < 0.001; and 130 [66.0%] vs. 52 [25.5%], *p* < 0.001, respectively) (Table 4).

**Table 4:** Outcomes by number of rib fractures as category according to the optimal cut-off

All study population	Number of rib fractures < 3 (*N* = 204)	Number of rib fractures ≥ 3 (*N* = 197)	*p*-value
Any intra-thoracic and intra-abdominal injuries	104 (51.0%)	175 (88.8%)	< 0.001
Significant intra-thoracic and intra-abdominal injuries	52 (25.5%)	130 (66.0%)	< 0.001
**Chest and upper abdominal trauma only**	**Number of rib fractures < 3 (*N* = 180)**	**Number of rib fractures ≥ 3 (*N* = 124)**	***P*-value**
Any intra-thoracic and intra-abdominal injuries	90 (50.0%)	106 (85.5%)	< 0.001
Significant intra-thoracic and intra-abdominal injuries	45 (25.0%)	76 (61.3%)	< 0.001

In multivariable logistic regression analysis, the number of rib fractures ≥ 3 (aOR, 3.01; 95% CI, 1.35–6.71; *p* = 0.007), lateral rib fracture (aOR, 3.94; 95% CI, 1.99–7.79; *p* < 0.001), and posterior rib fracture (aOR, 4.22; 95% CI, 1.99–8.97; *p* < 0.001) were independently associated with any intra-thoracic and intra-abdominal injuries. The number of rib fractures ≥ 3 (aOR, 2.97; 95% CI, 1.50–5.88; *p* = 0.002) and posterior rib fracture (aOR 3.02, 95% CI 1.65–5.54, *p* < 0.001) were independently associated with significant intra-thoracic and intra-abdominal injuries.

### Intra-thoracic injuries

In multivariable logistic regression analysis, age, requirement of O_2_ supplementation, number of rib fracture (aOR, 1.31; 95% CI, 1.09–1.57; *p* = 0.004), lateral rib fracture (aOR 3.38, 95% CI 1.69–6.78, *p* = 0.001), and posterior rib fracture (aOR, 2.45; 95% CI, 1.19–5.01; *p* = 0.014) were independently associated with any intra-thoracic injuries.

Requirement of O_2_ supplementation, number of rib fractures (aOR, 1.23; 95% CI, 1.05–1.45; *p* = 0.011), and posterior rib fracture (aOR, 2.76; 95% CI, 1.43–5.34; *p* = 0.003) were independently associated with significant intra-thoracic injuries.

### Intra-abdominal injuries

In multivariable logistic regression analysis, age, requirement of transfusion, lower rib fracture (aOR, 2.23; 95% CI, 1.01–4.94; *p* = 0.048), and posterior rib fracture (aOR, 1.74; 95% CI, 1.07–3.11; *p* = 0.042) were independently associated with intra-abdominal injuries.

### Subgroup analyses

In the subgroup conducted among those with only trauma to the chest and upper abdomen, requirement of O_2_ supplementation, number of rib fractures (aOR, 1.45; 95% CI, 1.12–1.89; *p* = 0.005), and posterior rib fractures (aOR, 2.62; 95% CI, 1.14–6.04; *p* = 0.023) were independently associated with any intra-thoracic and intra-abdominal injuries. Requirement of O_2_ supplementation, requirement of transfusion, number of rib fractures (aOR, 1.28; 95% CI, 1.05–1.57; *p* = 0.017), and posterior rib fracture (aOR, 2.05; 95% CI, 1.01–4.22; *p* = 0.049) were independently associated with significant intra-thoracic and intra-abdominal injuries (Table 5).

**Table 5:** Results of multivariable logistic regression analyses in the subgroup with trauma in the chest and upper abdomen only

	Any intra-thoracic and intra-abdominal injuries			Significant intra-thoracic and intra-abdominal injuries		
Variables	aOR	95% CI	*p*-value	aOR	95% CI	*p*-value
Sex (reference = female)	1.19	0.62–2.27	0.597	1.06	0.55–2.03	0.859
Age	0.98	0.96–1.01	0.158	1.00	0.98–1.03	0.785
High-energy trauma	1.70	0.89–3.23	0.105	1.69	0.91–3.14	0.099
**Initial vital signs**						
Systolic blood pressure	1.00	0.99–1.02	0.656	1.00	0.98–1.01	0.651
Diastolic blood pressure	1.00	0.97–1.02	0.764	1.00	0.97–1.03	0.957
Respiratory rate	0.96	0.89–1.04	0.373	0.92	0.80–1.05	0.217
Heart rate	0.99	0.97–1.01	0.278	1.01	0.99–1.03	0.350
**In-ED management**						
O_2_ supplementation	**3.72**	**1.75–7.92**	**0.001**	**4.39**	**2.34–8.25**	** < 0.001**
Mechanical ventilator use	0.00	0.00–Inf	0.988	0.32	0.01–10.61	0.520
Transfusion	5.60	0.42–75.04	0.194	**10.92**	**1.07–111.22**	**0.044**
**Rib fracture characteristics**						
Number of rib fractures	**1.45**	**1.12–1.89**	**0.005**	**1.28**	**1.05–1.57**	**0.017**
Bilateral rib fracture	0.44	0.13–1.45	0.176	0.59	0.20–1.73	0.338
Lower rib fracture	1.17	0.62–2.22	0.628	1.53	0.81–2.90	0.194
Displacement	1.51	0.53–4.32	0.442	1.44	0.61–3.23	0.380
Anterior rib fracture	0.51	0.17–1.48	0.214	0.74	0.28–1.96	0.545
Lateral rib fracture	2.16	0.92–5.04	0.076	0.70	0.32–1.55	0.384
Posterior rib fracture	**2.62**	**1.14–6.04**	**0.023**	**2.05**	**1.01–4.22**	**0.049**

Hosmer − Lemeshow test showed *p* > 0.05

*Abbreviation*: *aOR* adjusted odds ratio, *CI* confidence interval, *ED* emergency department

## Discussion

In patients with rib fractures due to blunt chest trauma, posterior rib fracture, lateral rib fracture, number of rib fractures, and requirement of O_2_ supplementation were independent factors for any intra-thoracic and intra-abdominal injuries. Posterior rib fracture, number of rib fractures, requirement of O_2_ supplementation, and requirement of transfusion were important factors for significant intra-thoracic and intra-abdominal injuries. The optimal cut-off for the number of rib fractures was three. The rib fracture characteristics mentioned above remained consistent in the subgroup with trauma to the chest and upper abdomen alone.

While previous studies included specific patients with specific locations of rib fractures, such as lower rib fractures [10, 11], our study included all patients with rib fractures due to blunt trauma. Furthermore, our study included patients with rib fractures with other injuries, which is more aligned to real-world conditions. In addition, we conducted subgroup analyses, whereas an earlier study only evaluated rib fractures with chest trauma [12]. The broader inclusion criteria make the results of our study more helpful in deciding on the necessity of a chest CT in the emergency department, compared with the results of previous studies. We performed a multivariable logistic regression analysis using variables that can be easily and rapidly obtained from physical examination, such as the location of the fracture and vital signs. All variables were obtained using noninvasive measurements. These factors can help emergency physicians decide to perform chest CT in the early stages of routine clinical practice.

Posterior rib fractures were associated with intra-thoracic and intra-abdominal injuries. The considerable direct force on the ribs causes a rib fracture and can also injure adjacent tissues [3, 13]. Posterior rib fractures might be an important factor for intra-thoracic and intra-abdominal injuries because the posterior component of the chest and abdomen consists of major arteries, such as the aorta and abdominal vessels, as well as the posterior part of the liver, spleen, lungs, and retroperitoneal organs. Therefore, patients with posterior rib fractures may require chest CT to evaluate organ injury.

Chest CT has been considered in patients who have undergone high-energy trauma mechanisms in previous studies [4, 5, 14]. However, trauma mechanisms were not independently associated with the outcomes in our study. As the chest wall of young patients is more elastic than that of elderly patients, rib fractures occur in high-energy trauma [3]. Meanwhile, rib fractures can easily occur in low-energy trauma patients with osteoporosis [13], which is more common in older patients than in younger patients [15]. This could be the reason why we identified age as an independent factor in intra-thoracic and intra-abdominal injuries, while the trauma mechanism was not. Additionally, the trauma mechanism was classified according to the patient’s self-report. The trauma mechanism might be inaccurate due to errors in recall or due to decreased consciousness or posttraumatic concussion. The amount of energy delivered to the ribs and adjacent organ might be more relevant to intra-thoracic and intra-abdominal injuries than the trauma mechanism. Thus, physicians should not decide to perform chest CT only based on the trauma mechanism.

The optimal cut-off of the number of rib fractures for the outcomes was three. This cut-off was similar to the widely used definition of multiple rib fractures [16]. In addition, this cut-off was suggested in a previous study of rib fractures and intra-thoracic pulmonary complications [12]. Sensitivity and specificity of previous study [12] were similar to those in our study. Furthermore, the presence of more than three rib fractures was independently associated with organ injury in a previous study [13] and our study. The number of rib fractures (especially if > 3) must be considered an important factor for intra-thoracic and intra-abdominal injuries.

A previous study evaluated intra-thoracic injuries such as pneumothorax, hemothorax, and lung parenchymal injuries and found that rib fracture displacement was associated with intra-thoracic injuries [17]. In a previous study, occult pneumothorax, scanty hemothorax, and small lung contusion might be included as outcomes, and the location of the fracture was not evaluated [17]. In contrast, displacement of the rib fracture was not associated with liver, spleen, or kidney injuries in a previous study [17]. The evaluation of significant intra-thoracic and intra-abdominal injuries and adjustment of multiple characteristics of rib fractures might lead to differences between the results of previous study and our study.

To evaluate the characteristics of rib fractures that did not require chest CT, we evaluated the factors associated with any injuries. However, clinically meaningful factors that could be used to omit chest CT were not found in the multivariable logistic regression analysis. Furthermore, trauma mechanisms and vital signs were not significantly associated with any injury. It is challenging to rule out intra-thoracic and intra-abdominal injuries without chest CT in the early stage in the emergency department. Therefore, a serial assessment of clinical findings is required to decide not to perform chest CT in patients with rib fractures.

This study had several limitations. First, due to the retrospective observational study design, covariables might have been missed. Second, this study was conducted at a single center. The characteristics of patients with rib fractures may differ between hospitals or trauma centers. Further multicenter or multi-trauma center studies are warranted. Third, this study evaluated the outcomes of the initial chest CT. Delayed organ injury due to blunt trauma may have occurred later. However, we believe that the proportion of delayed significant organ injuries is minimal. In addition, since this study was conducted at only a tertiary academic teaching hospital in one region, patients with delayed significant organ injuries might have revisited our hospital, but we did not observe any such cases in this study. Fourth, laboratory data were not included in the analysis. A previous study reported that the results of arterial blood gas analysis, such as pH and base excess, may be helpful factor [10]. In the present study, arterial blood gas analysis was not performed in a significant number of patients. Performing an arterial blood gas analysis might reflect the physicians’ expectation of severe injury, leading to selection bias. Fifth, diagnosis of rib fracture was based on chest radiography prior to chest CT and patients who did not undergo chest CT were excluded. Although there can be selection bias due to inclusion and exclusion criteria, most rib fracture patients (401/499, 80.4%) underwent chest CT and included in analysis. Soltanpour et al. reported that this practice does not always change the management of multiple trauma patients [18]. In addition, we performed analysis including rib fracture patients without chest CT and these patients were assumed to have no intra-thoracic and intra-abdominal injuries. The results were similar. Requirement of O_2_ supplementation, number of rib fractures (aOR, 1.51; 95% CI, 1.31–1.74; *p* < 0.001), lateral rib fracture (aOR, 1.88; 95% CI, 1.11–3.21; *p* = 0.020), and posterior rib fracture (aOR, 2.18; 95% CI, 1.26–3.77; *p* = 0.005) were independently associated with any intra-thoracic and intra-abdominal injuries. Requirement of O_2_ supplementation, mechanical ventilator use, requirement of transfusion, number of rib fractures (aOR, 1.45; 95% CI, 1.24–1.71; *p* < 0.001), posterior rib fracture (aOR, 2.10; 95% CI, 1.17–3.77; *p* = 0.013), and extremity fracture were independently associated with significant intra-thoracic and intra-abdominal injuries. We attempted to include patients similar to those observed in real-world conditions and performed an analysis using variables that can be easily, rapidly, and noninvasively obtained from routine clinical practice. The results of our study may help physicians in early decision-making to perform chest CT in patients with rib fractures in the emergency department.

## Conclusions

Among blunt trauma-induced rib fracture characteristics, posterior rib fractures, lateral rib fractures, ≥ 3 rib fractures or number of rib fractures, requirement of O_2_ supplementation are factors that indicate the need for chest CT to identify intra-thoracic and intra-abdominal injuries in the emergency department. The results of our study may help physicians in early decision-making to perform chest CT in patients with rib fractures in the emergency department.

## Data Availability

The datasets generated and/or analysed during the current study are not publicly available due to their containing information that could compromise the privacy of research participants but are available from the corresponding author on reasonable request.
